# A woman’s role in health leadership: an assessment of experiences during acute public health emergencies

**DOI:** 10.5365/wpsar.2024.15.5.1303

**Published:** 2025-11-24

**Authors:** He (Julia) Bai, Jocelyn J Herstein, Peta-Anne Zimmerman, Meru Sheel, Renée Christensen, Jocelyne M Basseal, Sharon Salmon

**Affiliations:** a College of Public Health, University of Nebraska Medical Center, Omaha, Nebraska, United States of America.; b School of Nursing and Midwifery, Griffith University, Gold Coast, Queensland, Australia.; c Infection Control Department, Gold Coast Health, Gold Coast, Queensland, Australia.; d Collaborative for the Advancement of Infection Prevention and Control, Gold Coast, Queensland, Australia.; e School of Public Health, The University of Sydney, Sydney, New South Wales, Australia.; f Sydney Infectious Diseases Institute, The University of Sydney, Sydney, New South Wales, Australia.; g Women in Global Health Australia, Camperdown, New South Wales, Australia.; h World Health Organization, Geneva, Switzerland.; i UNSW Medicine & Health, School of Population Health, University of New South Wales, Sydney, New South Wales, Australia.; j World Health Organization Regional Office for the Western Pacific, Manila, Philippines.

## Abstract

**Objective:**

We explored the experiences of women in senior or leadership roles in navigating and leading during acute public health emergencies.

**Methods:**

Women leaders in the World Health Organization Western Pacific Region attending the Global Outbreak Alert and Response Network’s Outbreak Response Leadership Training (11–18 September 2024) were invited to participate in this phenomenological study. Eleven interviews were conducted with training attendees and observational data were gathered. Inductive thematic analysis was conducted to identify key themes.

**Results:**

Four themes associated with women-centric experiences in public health emergency response were identified: disproportionate expectations in the workplace; the use of authoritarian decision-making during planning and implementation; encompassing different perspectives and leadership styles compared to men; and requesting additional opportunities and equitable prospects for career growth. Four themes that reflect non-gender-exclusive challenges experienced during emergency responses were also detailed. Themes observed were related to: barriers to efficiency; consequences of working within this field; and needs and necessities during emergency response.

**Discussion:**

This study highlights both gender-specific and systemic challenges experienced by women leaders in public health emergency responses, revealing how sociocultural norms and operational barriers intersect during times of crisis. We identified opportunities to assist women leaders through the recognition and promotion of different leadership styles, establishing a support network for women, and addressing systemic and organizational barriers that impact women.

Women have held a pivotal role in the global health sector as providers of clinical care, comprising 70% of all health-care workers, including over 80% of nursing professionals, over 90% of midwifery roles, and countless unpaid community health worker and caregiver positions. (*1*) Women in health leadership positions have been found to improve financial performance, encourage stability, increase engagement with environmental and health-focused initiatives and invest in worker welfare. (*2*) Such traits are essential within the context of an emergency, defined as “a situation impacting the lives and well-being of a large number of people or a significant percentage of a population and requiring substantial multisectoral assistance” and includes “humanitarian emergencies due to conflict, natural disasters, food insecurity, outbreaks and famine.” (*3*) In public health emergency response (PHER), emphasis is placed on the rapid distribution of tasks, flexible decision-making informed by evolving data and information, dynamic coordination among stakeholders, a knowledgeable and scalable workforce, and the redirection of resources to mitigate supply chain disruptions. (*4*)

Women leaders have demonstrated the ability to react decisively and lead with a more democratic and participative style that aligns with the communication and coordination needs of PHER, yet women have continuously faced inequities in the fields of emergency response and global health. (*2*, *5*) Documented gendered inequalities faced by women in PHER include inadequate or ill-fitting personal protective gear, elevated risk of exposure to infection, increased workload, greater economic insecurity, greater mental health burden and burnout. (*1*, *6*, *7*) Women leaders who operate within emergency response have also reported receiving less support than their male colleagues, both in technical areas such as epidemiological support and in non-technical areas related to administrative, communication or well-being assistance. (*7*) The lack of gender parity within all hierarchical levels of PHER-related sectors limits women’s ability to function to their full capacity, resulting in financial loss and harmful physical and mental health repercussions for both genders. (*8*)

The contrast between women’s role in health and their representation in health leadership illustrates an undeniable inequity known as the XX Paradox: women hold around 70% of the health worker jobs globally, yet they only constitute 25% of leadership roles in health. (*1*, *9*) From a representation perspective, women from low- and middle-income countries (LMICs) are least represented in global health leadership. (*1*) Although there has been advocacy on paper to seat women at the leadership table, informal rules and discrimination continue to exist against women at all stages of their professional life. (*6*, *10*) Women who pursue leadership positions often encounter gender-based obstacles during their career, such as sexual harassment, gender bias, discrimination, power imbalance, privilege and gender norms. (*9*, *11*)

It is crucial to understand the lived experiences, challenges and barriers faced by women leaders to identify, improve and provide adequate support for them in all roles during a PHER. Most research detailing gender and equity in the global health workforce has documented experiences from high-income countries that are not applicable to LMICs. (*9*) Although current knowledge has highlighted women’s roles in leadership, there is limited data sharing women’s perspectives on their experiences in leadership, particularly related to PHER.

Established by the World Health Organization (WHO), the Global Outbreak Alert and Response Network (GOARN) is a global technical partnership to strengthen and coordinate the rapid mobilization of experts responding to international outbreaks through outbreak response training, networking, research and collaboration. (*12*) Women leaders in the WHO Western Pacific Region have previously shared insights into their experiences, challenges, and advice for other women in PHER fields and roles. (*13*) A continuous evaluation of challenges and barriers is necessary to produce a temporal assessment of disparities for women within the field of PHER. Leadership programmes offer a critical space for networking and collaboration across diverse career levels and institutions. Further research evaluating the PHER-related experiences of women leaders is warranted to better explore the systemic barriers women face in PHER settings and address ongoing knowledge gaps, particularly across the Asia and Pacific region. (*13*) The objective of this study is to examine the experiences of women in senior or leadership roles among GOARN partners by identifying the key themes they rely on to navigate and lead during acute public health emergencies.

## Methods

### Study design

A phenomenological approach was used to capture data through in-depth, semi-structured interviews and participant observation. Phenomenology, a qualitative research approach that investigates the experience and perspectives of individuals around a certain phenomenon, has been used in prior literature to gain insight into the barriers and setbacks encountered by those in female leadership roles as they ascended into their positions. (*14*, *15*) An exploratory design was used for the interviews to investigate participant experiences and perceptions regarding their roles in PHER.

### Study population

Subjects eligible for the study included all 24 women who were invited to participate in the inaugural GOARN Outbreak Response Leadership Programme conducted in Darwin, Northern Territory, Australia, in September 2024. (*16*) Additionally, three mentors were invited to participate. Attendees were all senior-level women from GOARN partner institutions within the WHO South-East Asia and Western Pacific regions who had a minimum of 10 years of professional experience in a relevant field related to public health and significant outbreak response experience at international, regional and/or national levels.

### Recruitment and data collection

#### Semi-structured interviews

All participants were selected via purposive sampling and contacted individually by the WHO technical officer. To promote the inclusion of perspectives from all participating countries with representatives attending the training, participant outreach was based on country of origin. E-mails were sent with the participant information sheet detailing the objective and scope of the study. If interested in participating, invitees confirmed their availability and a 60-minute Zoom (version 6.3.11) interview time slot was scheduled for them by the interviewer. All interviews were conducted in English.

Interview questions were informed by available literature and related to challenges in emergency response, experiences surrounding leadership and women’s contribution to PHER. The interview questions are outlined in **Table 1**.

**Table 1:** Interview questions and probes of women’s leadership and contributions to PHER during the WHO Western Pacific Region GOARN Leadership Programme, 11–18 September 2024

Topic of focus	Question	Probe
**Career, responsibilities, accomplishments**	**Can you tell me about your current role in your organization?**	**What are some of the responsibilities and duties that you have?** **How long have you been in this role?** **What was your previous role right before this?** **Was this role recommended by a mentor?**
**Public health emergency response experience: challenges**	**What is the most challenging part about responding to public health emergencies?**	**Can you give me an example of this challenge?** **How did these challenges impact your ability to lead?** **How did you navigate those challenges?** **Did you receive any support for this challenge later on?** **What was the end result?**
**Public health emergency response experience: support and community**	**What types of support and resources have you found critical for public health response?**	**Do you wish you had more support?** **Do you think you and your male colleagues get different amounts of support?** **Where does the support come from?** **If individuals/peers: Are they female peers ** **as well?** **Were there any gaps in the support you received?**
**Peer-to-peer challenges**	**Have you had a negative experience with a peer while leading?**	**Can you tell me more about this experience?** **How did you overcome this issue?** **Do you think your gender played a role in this experience? Why or why not?**
**Leadership**	**Do you think women leaders contribute anything unique to public health emergency response?**	**Why or why not?** **If so, what are those things?** **How can our field better support women responders, from women at junior levels to those in senior positions?**

GOARN: Global Outbreak Alert and Response Network; PHER: public health emergency response; WHO: World Health Organization.

#### Workshop observation

All of the programme participants were subjected to naturalistic phenomenological observational data collection. (*14*) Investigators (JB, SS) observed each workshop seminar and recorded verbal and non-verbal cues and interactions between participants as field notes coupled with their own reflections of the observations. The investigators used their observation notes from the seminar to confirm intercoder consistency with the phenomenological concepts identified from the interviews. (*14*)

### Data analysis

Recordings of the interviews were transcribed verbatim using the Zoom transcription process and manually cleaned by comparing the transcript data to the Zoom recording. The transcript data were subjected to inductive thematic analysis, a six-step process of identifying themes and patterns within qualitative data by familiarizing oneself with the data, categorizing them into codes, then aggregating codes into major themes to determine significance of findings. (*17*) All transcripts and workshop observation notes were reviewed and evaluated by both analysis researchers (JB, JH) to reduce confirmation and selection bias before the interviews were independently coded. Disagreements over the categorization of codes or themes were resolved through discussion among analysis researchers until consensus was reached. All analyses were conducted in NVivo 12 Pro.

## Results

Of those invited, interviews were conducted with 11 individuals (10 participants, one mentor) from eight countries and areas: Australia, Cambodia, Fiji, Guam, Indonesia, Singapore, Tonga and Viet Nam. Australia had three participants and Singapore had two participants; all other countries were represented by one participant. Participants were between the ages of 35–65 years and held a wide range of professions, including medical physician, health-care organization director, epidemiologist, veterinarian and laboratory clinician. All interviews were conducted on 11–18 September 2024, with an average interview time of 55 minutes (range 30–61 minutes). Through thematic analysis, four themes associated with women-centric experiences in PHER and four themes related to the overarching PHER experience were identified and reported in **Table 2** and **Table 3**, respectively.

**Table 2:** Women-centric experience in public health emergency response themes identified during interview questions and probes of women’s leadership and contributions to PHER during the WHO Western Pacific Region GOARN Leadership Programme, 11–18 September 2024

Theme	Code	Code explained	Example quote
**Shattering glass cliffs, dismantling glass walls** **Struggles for women to have their voice heard among male peers** **Gender imbalance within the workforce** **Disproportionate need for women to build credibility and prove their worth in their field** **Placing women in leadership positions during times of high risk**	**Glass wall**	**Pertains to struggles for women to have their voice heard and to maintain the same level of open dialogue among their male peers as male peers would among each other. Communication is paired with credibility for women, often through what is acceptable in terms of communication and leadership style.**	**“They will support you sometimes and they will tell you that you can do this, and you cannot do that. But if the is [sic] team integrated together, male and female, then male is the priority.” (P2)**
	**Glass cliff**	**Instances of placing women in positions of leadership during times of high risk in emergency response, where women could be subjected to greater criticism or scrutiny.**	**“I think also, when it comes to, say, doing extra work or doing extra coverage because there was a surge or something, sometimes it seems it's the same people who are asked again, the women tend to be asked because maybe we won't say no.” (P5)**
	**Boy's club**	**Discussions emphasizing the gender imbalance within the workforce in certain countries and the disproportionate need for women to build credibility to prove their worth in their field compared to their male colleagues. While some countries exhibit a male majority in senior positions, others have male-dominated workforce in all levels of public health. Female participants from Asian countries, in particular, have emphasized feeling outnumbered.**	**“…Particularly in Asia, most of the higher positions are held by males and for the females, not only you have to proof [sic] that you are more than capable, but you also have to find ways to be able to break yourself into this so-called ‘male domain’ and be able to fully understand their mental models and how be a part of them.” (L2)**
**Owning authority** **Women’s roles as active, responsive leaders** **Having to stand their ground on decisions made by senior positions or external collaborators** **Importance of trust and honesty in messaging during emergency response**	**Standing firm**	**Difficulties in having to push back or having to stand your ground on decisions made by senior positions and external collaborators during emergency response.**	**“I know one meeting I got out [sic] and I think one of the executive directors said to me, ‘You know, we usually try to be on the same page’, and things like that, and I said, ‘Well, you don't pay me to be on the same page. I think you're wrong’.” (P1)**
	**Messaging during emergency response**	**The impact of trust and honesty in messaging among public health partners and the community. Codes detail how lack of trust in messaging can become a barrier in emergency response; alternatively, trust and honesty in messaging helps guide policy changes that impact response. Messaging with the public must be paired with honesty and transparency for trust to remain.**	**“I just had to keep being honest and repeat myself saying, ‘Yes, this is a situation and we need your help. We can't do it alone, but if you don't want to take these preventive measures, correct our course’ […] trying to be out there first [as the] first voice. Be honest with what you know, what you don't know, and then come back and give updates frequently. So those kinds of things, be present.” (P7)**
	**Active leadership potential**	**Instances of placing women in positions of leadership and having an active, responsive leader who is open to discuss and dissect issues.**	**“…It's more direct now, whenever we come in with a request. She's been trying to address them immediately compared to the former heads for public health, I think, in terms of meetings and discussions. There's more opportunity for us to discuss and dissect any issue with our female leader at the moment compared to the previous leader.” (P11)**
**Cultivating resilience and shaping perspective** **Resilience and tenacity to operate in public health workforce** **Women encompass a broader perspective** **Practice adaptability** **More approachable, more empathetic** **Emotional awareness**	**Resilience and perspective**	**Women have the resilience and strength to persevere due to having to do so in their personal lives. Women also hold a broad perspective and can look at tasks with a more empathetic lens compared to men. Highlights the strength in using emotions instead of viewing it as a weakness.**	**“We can be more empathetic. We bring our emotions in, and some people will be like, no, that's not good, but you have to always remember there's the human side of any emergency response, right?” (P7)**
**Creating equitable opportunities** **Additional opportunities for women to experience leadership** **Equitable prospects** **Connections with women role models and mentors** **Network of support**	**Equitable opportunities**	**Discussions pertaining to women needing additional support and networking with other women leaders. Mentorship should be bidirectional. Providing more opportunities for women in senior positions would be beneficial for promoting leadership among all levels of government.**	**“I think that the junior responders need more support than the senior responders. The first engagement, I think, support in terms of having someone to talk to is important and with the senior responders, it's supporting the decision-making processes.” (P12)**

**Table 3:** Overarching public health emergency response themes identified during interview questions and probes of women’s leadership and contributions to PHER during the WHO Western Pacific Region GOARN Leadership Programme, 11–18 September 2024

Theme	Code	Code explained	Example quote
**Leading under emergency response pressure** **Burnout, mental fatigue** **Support seen among ER departments** **Asking for help could be considered taboo** **Nonstop pressure to perform** **Guilt from feeling fatigue**	**Mental health and fatigue**	**Comments pertaining to burnout and mental fatigue during ER, prioritizing work while placing all other aspects in one’s life secondary. Discussions of how public health sectors have supported mental health during ER, from support groups to taking physical leave from the stationed post. Asking for help while working in ER could be considered a taboo subject for some.**	**“It's hard to know yourself when you're fatigued when you're just wanting to get the job done so I think that's probably something that leaders need to [keep] a closer eye on and not just work their teams to the absolute end of their energy level, but to manage teams [with] taking consideration of breaks and not just having a break and then everyone go out drinking.” (P14)**
	**Pressure to perform**	**Having nonstop pressure to continuously perform, with feelings of guilt and need to overwork considered the norm. Furthermore, engaging with new colleagues and team dynamics while simultaneously trying to keep up with tasks and implementation of strategies during acute ER while new information could be dropped at any time.**	**“I’ve done so many interviews, and I was at a point where I've had enough and I thought I have to say no to these things, and my communications person called me after I declined something and she said, ‘you did this, really well and as a woman in science, I want you to do this’[...] This was my breaking point, just leave me alone, why can't I just do my job? Get someone else to do that interview? And at that point, I don't care, I'm just a scientist. Take another scientist. I don't want to do everything that's there to represent women. Just go away.” (P16)**
**Underscoring priorities during emergency response** **Collaboration among internal and external partners is essential** **Pivoting strategies and tasks during ER based on updated information** **Working on ER simultaneously with daily tasks** **Prioritization of tasks based on hierarchy of urgency related to ER**	**Multidisciplinary collaboration**	**When having multidisciplinary teams, priorities can differ among colleagues and may require additional deliberation and communication. One barrier discussed is the lack of prior established collaboration or networking within internal and external partners, leading to lengthening time for follow-up, identifying personnel for tasks, etc.**	**“So communications become a little bit steep, because [it is no] longer somebody that you know very well. Coordination also became a little bit of the issue because you need to work with them in terms of what priorities would be. And your priorities and their priorities may not necessarily be the same, because we have our own agenda depending on who you work with and the issues at hand.” (L2)**
	**Pivoting decision-making**	**Mentions of pivoting public health suggestions during ER due to the most updated evidence and research, leading to changes in the prioritization of resources and funding as well as changes in policy.**	**“The anecdotal evidence is important but so is generating our own research and also taking into account research done across the world, whether it's the recovery trial or other studies, and then using that evidence to pivot your policy in a mindful way.” (P5)**
	**Adjusting work with ER duties**	**Impact of having to modulate work outside of ER and private life with ER-related duties, including excess travel, hierarchy of 'urgency' in simultaneous outbreak response, and realizing when an unfolding outbreak becomes severe and requires more attention and priority.**	**“I look at other people and think, how are they like so calm. And everything will be going on and then part of my team will come in and I'm like, I don't have time for this. I know that I need to moderate that response because I can't be seen as out of control or that you don't have the situation under control, but I'm not really a calm person, so that I feel like under time pressures, it just becomes more evident.” (P16)**
**Overcoming capacity barriers pre-response** **Guidelines need to be revised and updated** **Cascading issues due to outdated regulations and guidelines, lack of resources and workforce, etc.** **Multilevel mobilization during ER can cause delay in tasks** **Requiring additional workforce and training before ER**	**Outdated guidelines**	**Details outdated government regulations, procedures, and newly created emergency guidelines and how they impact ER preparedness and efficiency. Outdated guidelines pertaining to ER are mentioned to cause cascading issues with providing efficient workforce and funding as well as understanding expected roles and duties during ER.**	**“We have the law [regarding infection control] but is over 20 years with no adjustment, so the emergency operation center is not included. Because emergency response is not included in the law, in many states, parties are not clear about their roles in emergency, and then they are not active in their response.” (P13)**
	**Multilevel infrastructure mobilization**	**Discussions involving the necessity of multilevel government mobilization in ER and how government infrastructure can impact timeliness and decision-making when it comes to acute emergency responsiveness and policy.**	**“We [would like to] take care of our own procurement and recruitment because there's delays, we’re talking months, years for some things like recruitment. We've been trying to recruit some positions for years and we still haven't gotten anyone because we’re dependent on [another department’s] personnel section to do that, but they also have more than 30 other agencies they have to assist.” (P7)**
	**Lacking resources and logistics**	**Comments regarding lack of resources and funding that led to shortages of equipment, testing capabilities, facilities for suspected patients, etc. A lack of pre-emergency response training and logistics can further cause delays during ER.**	**“In this year, 2024, there was a lack of the budget for testing and that made it more difficult to do the proper testing when we have cases in the field. So, I really hope in 2025, we will get more support...” (P9)**
	**Lacking workforce capacity**	**Mentions a lack of technical experts, workforce, and issues concerning the migration of personnel from the workforce completely, leading to necessary procurement of replacement personnel and additional training.**	**“The main problem that I encounter is our workforce capacity. We had few technical experts on the ground. We definitely need technical assistance from abroad.” (P11)**
	**Additional training**	**Comments regarding training and exercises needed before ER to increase knowledge in emergency management for within-country response and deployment. Training will promote adaptability during ER, familiarity with responsibilities and duties, confidence, and enhance decision-making.**	**“I think increasing their knowledge in public health emergency management. We can do some training that is some emergency, or some outbreak [under] different contexts.” (P13)**
**Mobilizing community support** **Collaboration between community and public health partners to establish partnerships during ER** **Finding support and trust among colleagues and team members** **Coming together with information from different departments and teams, disseminating details related to ER**	**Community outreach**	**Encouraging engagement with members of the community to participate in certain aspects of ER that include correspondence with the community they are a part of. In doing so, ER personnel and the community collaboration will further bridge partnerships and trust.**	**“So that's how I picked up that we really need help from the people in the community, and we were able to do contact tracing training with non-health participants. We identified some supporters from the community, and we conducted training on contact tracing with them.” (P11)**
	**Colleague support**	**Discussions involving finding support among colleagues and team members during ER. Comments reflect sharing perspectives on an issue, communicating and piecing together what each team member is working on independently, creating new networking opportunities, and working collaboratively.**	**“Maybe when I'm become a leader and I have staff or colleagues to do the program together, they can freely to ask me [if they don’t understand me] and they can tell me whatever I said that bothered them.” (P9)**

GOARN: Global Outbreak Alert and Response Network; ER: emergency response; L: leader/mentor; P: participant; PHER: public health emergency response; WHO: World Health Organization.

**Table:** 

GOARN: Global Outbreak Alert and Response Network; L: leader/mentor; P: participant; PHER: public health emergency response; WHO: World Health Organization.

### Women-centric experiences in public health response

Themes pertaining to women-centric experiences in the field of PHER highlighted gender-specific challenges faced by women leaders and how they navigate their responsibilities despite the obstacles they encounter. The themes capture the sociocultural influence on the workplace during PHER and the barriers it creates for women in leadership positions. These themes included: 1) shattering glass cliffs; dismantling glass walls; 2) owning authority; 3) cultivating resilience and shaping perspective; and 4) creating equitable opportunities.

#### Shattering glass cliffs, dismantling glass walls

This theme emphasized the gender imbalance within the workforce and the disproportionate need for women to build credibility and prove their worth within their PHER field. Participants discussed instances of the “glass cliff” theory, when women are placed in positions of leadership during times of high risk and are subjected to greater scrutiny. (*18*) Women also shared their struggles in having their voice heard, their communication style accepted, and maintaining open dialogue with their male peers compared to that between male peers. Contrasting perspectives were observed among Pacific island participants, who indicated feeling heard and accepted among their male colleagues, attributing it to the matriarchal aspects within their culture.

#### Owning authority

This authoritative theme highlighted women leaders providing an active, responsive role in PHER. Participants discussed the importance of standing firm on decisions when planning and implementing action plans with senior collaborators and external partners. The importance of trustworthiness and honesty in messaging between public health partners and the community was emphasized.

#### Cultivating resilience and shaping perspective

Participants characterized women leaders as empathetic and approachable due to their emotional awareness. They reported that women have a different, broader perspective compared to men, often practising adaptability and incorporating wider considerations into their work as opposed to lateral thinking during PHER. Participants recounted using these qualities while communicating with colleagues and the community.

#### Creating equitable opportunities

Additional opportunities for women to experience leadership and equitable prospects for lateral career growth were identified as a necessity. Participants reported that women role models and mentors are important for shaping a network of support for women in all chapters of their career journey. Some participants further indicated that active support from male mentors has been helpful in progressing their career within male-dominated workforces.

### Overarching public health emergency response experience

Themes related to the overarching PHER experience reflected in this section include the challenges related to emergency response that are not gender-exclusive and the barriers to efficient emergency response. Participants also illustrated the consequences of working within the PHER field and their needs and necessities during response. Four themes emerged here: 1) leading under emergency response pressure; 2) underscoring priorities during emergency response; 3) overcoming capacity barriers pre-response; and 4) mobilizing community support.

#### Leading under emergency response pressure

This theme focused on the topics of mental health, fatigue and the pressure participants feel to work past their capacity. They reflected on the hypocritical assertion that seeking help is considered taboo while overworking is praised and expected, highlighting a cultural contradiction. Responses urge for prioritized monitoring of workers’ mental health during PHER.

#### Underscoring priorities during emergency response

This theme discusses finding space for PHER among daily tasks and obligations, pivoting PHER recommendations and tasks based on updated evidence and research, and difficulties related to multidisciplinary collaboration in PHER. Participants identified a lack of network or established multidisciplinary teams as a barrier to timely PHER management while dealing with normal business.

#### Overcoming capacity barriers pre-response

Participants discussed many systemic concerns that impact PHER, including outdated guidelines and regulations that require revision to reflect current PHER standards, bureaucratic delays, lapses in resources and workforce shortages. Participants emphasized the importance of logistics preparation, pre-response training and the strengthening of in-country health emergency workforce capacity.

#### Mobilizing community support

The community support theme highlights participants’ comments detailing the need for partnerships with community members to participate in PHER activities that further encourage trust and transparency. Participants discussed collaboration during PHER, relying on colleagues for support, expert-led decision-making, communicating the latest developments and establishing network partners.

## Discussion

This study highlights both gender-specific and systemic challenges experienced by women leaders in PHER, revealing how sociocultural norms and operational barriers intersect during times of crisis. Through the themes identified in this study, participants illustrated the complexity of navigating PHER roles while striving for trust, adaptability and meaningful collaboration.

Current systems and policies used in global health security and PHER are predominantly created by men from high-income countries, resulting in a lack of collaborative engagement with women leaders, specifically from LMICs. This results in failure to consider the disproportionate impact that outbreaks have on the predominantly female global health workforce. (*19*) With the participation of GOARN-associated women leaders, we were able to capture the unique challenges experienced in LMICs through the immersive, multifaceted training programme.

Patriarchal culture, gender stereotypes and gender-based expectations affecting women in the workforce were noted to be significant among participants from LMICs in our study. Gender, along with seniority, come with pre-established biases about the capabilities, usefulness and trustworthiness of women. Traditional gender ideology, which emphasizes the role of men in the workforce due to being the sole income earner in the household, creates workplace gender disparities, leading to the sidelining of women’s input and the promotion of a disproportionate need for credibility among women in exchange for trust. (*20*) During times of crisis in PHER, the “glass cliff” effect places women in the role of the “miracle worker” at the forefront of the firing line, often burdened with relentless requests and a small margin for error, as indicated by our interviews. (*18*) However, when placed in positions of leadership, women’s ability to wield both stereotypically feminine and masculine behaviour during PHER simulates assertive but humane executive authority. (*21*, *22*) Similarly, participants remarked how the stereotypically masculine qualities of decisiveness and assertiveness are required during PHER when priorities differ among competing stakeholders and resources are limited. Participants also viewed their stereotypically feminine qualities of empathy and compassion positively, using their emotions to provide a communal sense of shared goals during multidisciplinary collaboration. Such prioritization of anticipatory policies and preventive measures seen among women leaders builds the capacity and resilience needed during PHER. (*21*)

The issue women face within the global health workforce is double-barrelled. One, women are consistently underrepresented in PHER and global health leadership with structural, systemic and social barriers that continue to present substantial obstacles against career advancement. (*23*-*25*) Two, the act of including women within established processes and systems without considering gender-responsive leadership will reinforce the persistence of gender-based discrimination, pay gaps and other workforce disparities. (*26*, *27*)

### Implications

Through our study, we identified improvements to assist women leaders during PHER and promote the future of women in leadership positions.

The most obvious strategy to mitigate gender disparities in PHER leadership is through the promotion of gender mainstreaming (gender-focused evaluation of all planned action, policies and processes) for leadership roles, further reducing gender-based expectations and biases regarding competency, capabilities and responsibilities. (*28*) One way of doing so is to recognize and promote leadership styles and qualities that are different from stereotypically masculine qualities associated with hierarchical, directive leadership styles. (*29*) The evaluation of women’s leadership competence is more directly related to their communal behaviours than their male counterparts. (*30*) Similarly, displaying traditionally masculine leadership qualities evokes greater penalties for women regarding leader likeability, while these qualities displayed by male leaders are associated with greater likeability. (*30*) By challenging gender stereotypes in leadership, leadership can be evaluated based on merit rather than gendered expectations.

More equitable opportunities for leadership positions, fostering mentorship among women leaders and establishing a network for women to connect could reduce feelings of isolation and cultivate support from women in similar positions. Challenging organizational structural barriers through evidence-based interventions rather than leaning on professional development to prepackage women to fit leadership roles will instead promote female representation at an institutional level, not the individual level. (*31*) Daily reflections from all participants of the leadership training supported GOARN’s objective of establishing a network of women leaders within the field of PHER and fielding mentorship opportunities among them. Furthermore, providing leadership training to all workplace leaders can help ensure that gender-mainstreaming policies and procedures are consistently understood and that gender-related responsibilities are more evenly shared.

In addressing overarching PHER themes, greater emphasis should be placed on reducing fatigue and burnout and prioritizing a proactive approach towards mental health. Women often have a greater risk of disease exposure, an increase in workload and elevated emotional burden due to guilt and a heightened sense of duty for community and patients. (*32*) The promotion of proactive mental health support among PHER workers can strengthen resilience against burnout, improve workforce retention and support teamwork efficiency and morale. (*33*) Also, addressing systemic and organization-level contributors to burnout and fatigue through intentional changes to policies and practices can provide actionable intervention while providing benefits to the employer as well. (*34*, *35*) Paid leave, subsidized childcare and eldercare, and flexibility in scheduling can further increase job retention and ensure productivity and focus. (*34*, *36*)

Participants from some countries requested that in-country capabilities through technical training, stakeholder collaboration and the updating of PHER guidelines be prioritized; specifically, that women’s differential needs be addressed due to their overwhelming contributions to front-line health care and PHER. Given the lack of gender-specific language among current PHER management and planning, future training and guideline revisions should include gender-inclusive frameworks to ensure women PHER workers are supported and compensated. (*33*) The revision of guidelines and procedures will also ensure PHER is managed with updated methodology and streamlined emergency response procedures, thereby enhancing equity and reducing waste by optimizing resource and workforce allocation. (*32*) The latter is especially important for countries that require stronger community resilience due to limited in-country resources and workforce capacity. Additional training among PHER workers will enhance response time, improve efficiency, foster proactive multilevel decision-making and encourage collaboration among stakeholders. (*37*)

### Positioning and reflexivity

As researchers and women with a background in emergency response, we acknowledge that our interpretation of the data is reflected by our personal lived experiences. We adopted insights and observations from existing literature and encouraged ongoing team discussions to mitigate the subjectivity of our observations. We aimed to provide transparency and encourage our audience to critically assess our findings within the context of our positionalities.

### Limitations

There were some limitations. Our recruitment was opportunistic and purposive and, hence, the findings cannot be generalized. There were participants from other countries in the programme who did not participate in our study and may have contributed additional perspectives to our research. Similarly, participants who attended the GOARN programme may not be representative of PHER leaders who did not attend. Participants were asked to retrospectively reflect on details related to PHER, subjecting our research to potential recall bias. The power dynamics associated with leadership positions may produce bias when recalling events. Furthermore, our single mentor interview may produce outlier data due to their elevated leadership position in comparison to other participants. Transcription data were manually reviewed, providing opportunity for human error during the data cleaning phase. There is a risk of data saturation and bias with coding reliability due to the limited number of researchers involved in the assessment and data categorization. Personal and cultural aspects of behaviour and belief were not prioritized during the interview, both of which could impact perspective. Finally, the limited number of attendees and publicly available communications materials regarding the training programme can lead to a risk of participant identification. We aimed to reduce this risk by de-identifying personal details from their interview responses, including their organization, specific leadership titles and country of origin.

### Conclusion

Women’s achievements and contributions to the field of PHER have been extensive, despite the inequities, challenges and barriers they have faced. Our study highlights the lived experiences of women leaders in PHER by capturing the lessons learned both in the field and throughout their careers. Our findings emphasized differing societal and cultural disparities that continue to impact women of all PHER fields. We hope that by encouraging equitable opportunities in leadership positions, in-country capacity-building and the implementation of mental health support interventions will enhance PHER practices and empower respondents in all positions.
